# Switching Failure Mechanism in Zinc Peroxide-Based Programmable Metallization Cell

**DOI:** 10.1186/s11671-018-2743-7

**Published:** 2018-10-19

**Authors:** Firman Mangasa Simanjuntak, Sridhar Chandrasekaran, Chun-Chieh Lin, Tseung-Yuen Tseng

**Affiliations:** 1 Department of Electrical Engineering, National Dong Hwa University, Hualien, 97401 Taiwan; 2 Department of Electrical Engineering and Computer Science, National Chiao Tung University, Hsinchu, 30010 Taiwan; 3 Department of Electronics Engineering and Institute of Electronics, National Chiao Tung University, Hsinchu, 30010 Taiwan

**Keywords:** Resistive switching, Programmable metallization devices, Zinc peroxide, PMC

## Abstract

The impact of peroxide surface treatment on the resistive switching characteristics of zinc peroxide (ZnO_2_)-based programmable metallization cell (PMC) devices is investigated. The peroxide treatment results in a ZnO hexagonal to ZnO_2_ cubic phase transformation; however, an excessive treatment results in crystalline decomposition. The chemically synthesized ZnO_2_ promotes the occurrence of switching behavior in Cu/ZnO_2_/ZnO/ITO with much lower operation current as compared to the Cu/ZnO/ITO (control device). However, the switching stability degrades as performing the peroxide treatment for a longer time. We suggest that the microstructure of the ZnO_2_ is responsible for this degradation behavior and fine tuning on ZnO_2_ properties, which is necessary to achieve proper switching characteristics in ZnO_2_-based PMC devices.

## Background

The volatile dynamic random access memory and non-volatile flash memory have been the main leading devices for data storage application in the market; however, their further development has reached their physical limits [1, 2]. Recently, programmable metallization cell (PMC), a class of resistive random access memory (RRAM), has attracted considerable interest due to its potential for the future data storage application [3–5]. A PMC device consists of a two-terminal sandwich structure which has the advantage of the high scalability and simple fabrication [3–7].

ZnO is one of the most popular materials for various electronics; due to its low cost, non-toxic, chemically stable, low synthetic temperature, and simple fabrication process [8]. Its direct band-gap of ~ 3.3 eV makes ZnO as a suitable candidate for transparent electronic devices [9–12]. However, up to now, the ZnO-based PMC devices still need to overcome many challenges which inhibit its realization. One of the main problems is that the ZnO-based PMC devices often require high operation current due to the high n-type conductivity of ZnO material [8]. PMC device having a high-resistive storage layer is compulsory to produce switching characteristics at low operation current. Several methods have been developed to alter the switching characteristics in ZnO-based PMC devices; such as, by introducing a dopant(s) [13–18], controlling the film growth [19, 20], adding a buffer or barrier layer [16, 21], inserting a nanorod layer [22, 23], and stacking with another material(s) [24, 25]. However, those approaches still require a complicated and time-consuming fabrication process.

Recently, we reported that the employment of zinc peroxide (ZnO_2_) layer in PCM cell exhibits volatile and non-volatile switching characteristics [26]. A peroxide surface treatment on ZnO surface may transform ZnO hexagonal into ZnO_2_ cubic phase [27–37]. The ZnO_2_ phase is found to have superior resistivity; thus, it can be exploited for Schottky contact and photodiodes applications; however, the potential of ZnO_2_ for switching memory, especially the switching characteristics modulation by controlling peroxide treatment is still less investigated [26, 29–38]. Therefore, a detail investigation on the impact of peroxide surface treatment on switching characteristics is necessary for further adoption and realization of ZnO_2_-based switching memory.

## Methods

ZnO thin film was deposited onto a commercial ITO/glass substrate (purchased from Uni-onward Corp.). The deposited films were immersed in hydrogen peroxide (30% H_2_O_2_, Perkin Elmer) solution at 100 °C for 1, 3, and 9 min. Hereafter, the surface-oxidized films were rinsed and dried with DI water and an N_2_ gas gun, respectively. In order to fabricate Cu/ZnO/ITO sandwich structure devices, Cu top electrodes with a diameter of 150 μm were sputtered onto the samples (patterned using a metal shadow mask). On a separated experiment, non-surface-treated film (NT) was prepared as a control sample for comparison. STx was used for denoting surface-treated samples, where *x* is 1, 3, and 9 representing the treatment time (minute), respectively. Crystal structure and morphology of the films were investigated using a transmission electron microscopy (TEM, JEOL 2100FX). A semiconductor device analyzer (B1500, Agilent Tech. Inc.) was used to study the electrical characteristics.

## Results and Discussion

**Fig. 1:**
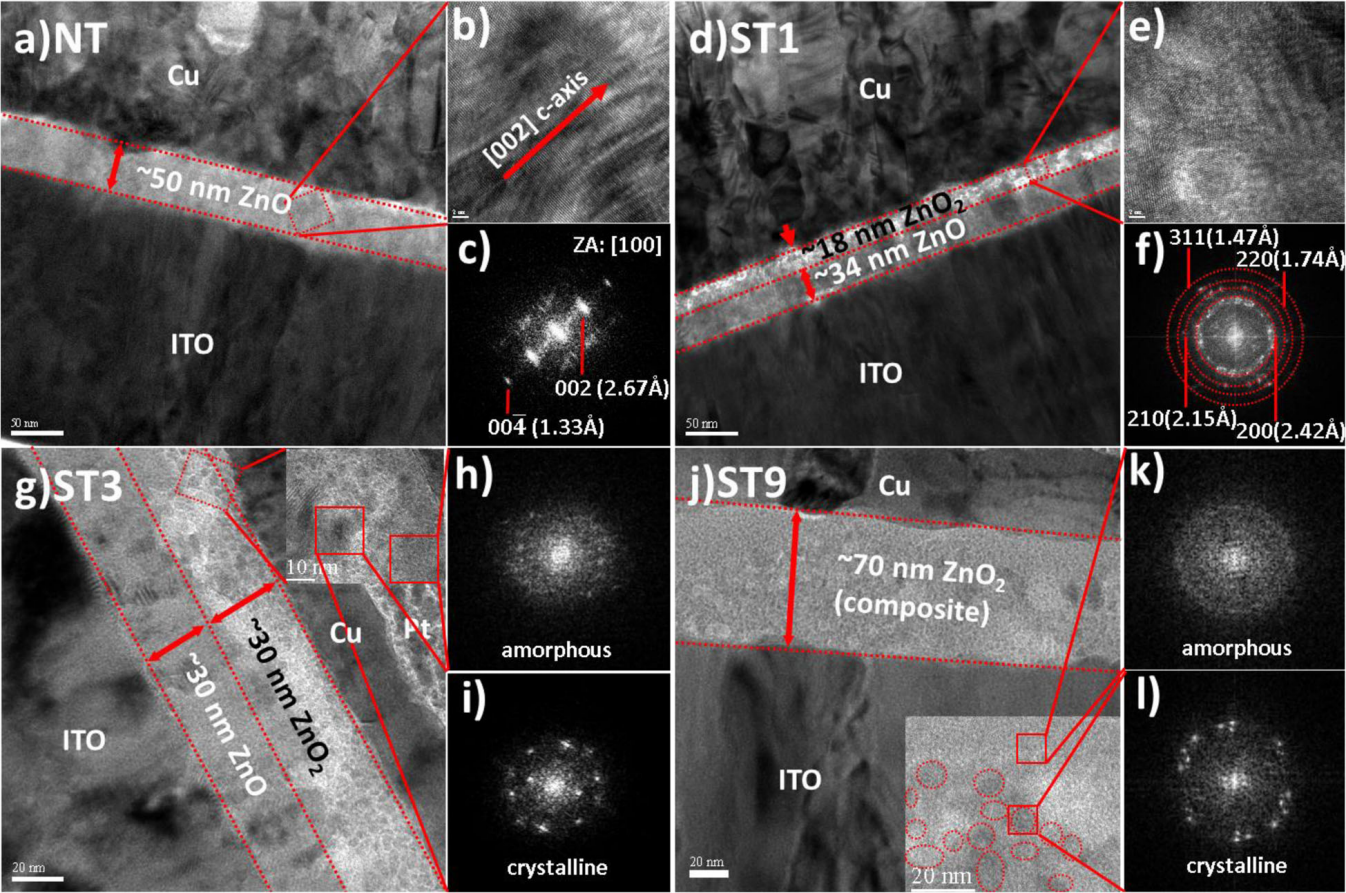
TEM analysis of (**a**–**c**) control, (**d**–**f**) ST1, (**g**–**i**) ST3, and (**j**–**l**) ST9 layers. The inset in (**g**) and (**j**) are high-resolution TEM images of ST3 and ST9, respectively

TEM analysis was conducted to investigate the effect of peroxide treatment on the structural and morphology of the films. Figure 1a shows the cross-sectional image of ZnO film (NT) grown on ITO substrate. It is found that the growth orientation of the film is perpendicular to the substrate as shown in the high-resolution (HR) TEM image in Fig. 1b.The crystal structure of the film was investigated by analyzing the fast Fourier transform (FFT) micrograph of Fig. 1b, as depicted in Fig. 1c. The crystal structure of the ZnO film is hexagonal wurtzite structure (match with JCPDS#36-1451). The structure and morphology of the surface of the ZnO film are altered after peroxide treatment for 1 min (ST1), as shown in Fig. 1d. It can be seen that the treatment leads to a formation of a double layer. The preferred (002) orientation is diminished in the upper layer, as shown in Fig. 1e; which indicates that phase transformation is occurred due to the peroxide treatment. Figure 1f shows spot pattern analysis of FFT micrograph of (e). The upper layer is found to be polycrystalline cubic pyrite structure ZnO_2_ (match with JCPDS#77-2414). It is confirmed that peroxide treatment induces hexagonal-to-cubic (h-to-c) phase transformation; this phenomenon corroborates with the previous literature [27, 28]. A peroxide treatment for 3 min (ST3) may lead to further oxidation into the deeper region, as depicted in Fig. 1g. The transformed region increases the total thickness of the resistive layer. The inset in Fig. 1g shows the HRTEM image of the transformed region. The FFT micrograph analysis shows that some small area has been transformed into the amorphous phase, as depicted in Fig. 1h and i. As the treatment time increases to 9 min (ST9), the phase transformation occurred in the whole region of the resistive layer, as shown in Fig. 1j. Consequently, the resistive layer consists of a single layer structure with an increased thickness of 70 nm. The inset in Fig. 1j shows the HRTEM image of the resistive layer. It can be observed that the resistive layer consists of a random distribution of nano-sized crystalline ZnO_2_ particles in the amorphous matrix, as confirmed by FFT micrographs analysis shown in Fig. 1k and l. This suggests that an extended peroxide treatment may lead to a crystalline decomposition. We suppose that the excessive oxygen radicals diffused into the crystalline material may destruct its crystal structure, thus transformed into the amorphous phase [28, 39]. The electrical measurement was carried out in order to evaluate the influence of the peroxide treatment on the resistive switching characteristics.

**Fig. 2:**
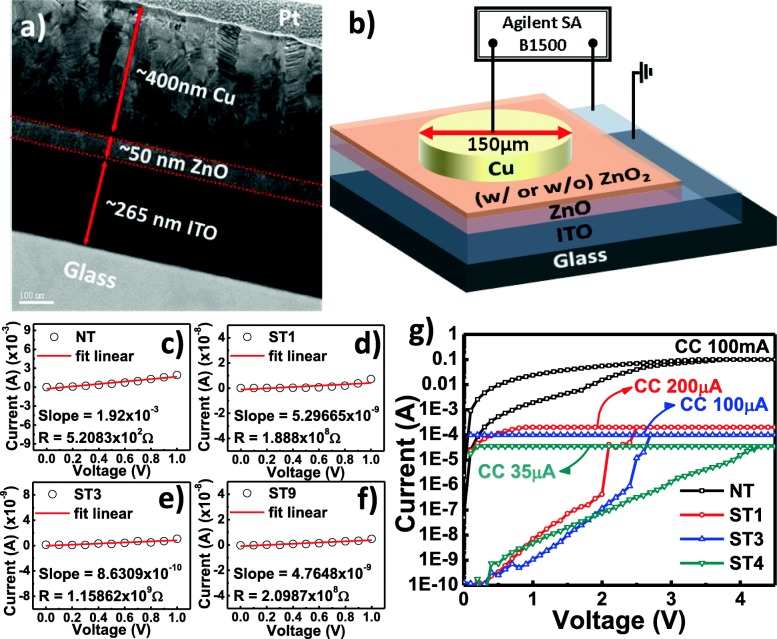
**a** Cross-section TEM image of Cu/ZnO/ITO device. **b** Schematic of Cu/ZnO/ITO device. Typical I-V curve and resistance calculation of (**c**) control, (**d**) ST1, (**e**) ST3, and (**f**) ST9 devices. **g** Forming curves of the fabricated devices

Figure 2a shows the cross-sectional TEM image of the fabricated control (NT) device. The thickness of the top electrode (Cu), resistive layer, and bottom electrode (ITO) is approximately 400, 50, and 265 nm, respectively. ITO bottom electrode was intentionally chosen due to the ZnO/ITO ohmic contact behavior [28, 36]; thus, the switching characteristics solely rely on the resistivity of the switching layer. The schematics of the device structure and measurement setup are depicted in Fig. 2b. The bias voltage is applied to the top electrode while the bottom electrode is ground. It is reported that the ZnO_2_ possesses a very high resistivity, due to the annihilation of intrinsic donor defects and formation of acceptor defects during peroxide treatment [28–30, 32, 35, 37]. A low-voltage sweep test is conducted to calculate the resistance of the pristine devices, as shown in Fig. 2c–f. It is found that the devices made with ZnO_2_ layer exhibit an increased pristine resistance, for up to 6 to 7 order of magnitude as compared to the device without the ZnO_2_ layer (control device). An excessive peroxide treatment (9 min) resulted in a slight decrease in resistance of the ST9 device (Fig. 2f). Previous studies suggest that the decrease in resistance after an excessive peroxide treatment is probably due to microstructural damage such as partial etched and surface roughing [35, 37]. However, such surface damage was not observed in our TEM analysis. Nevertheless, the formation of the amorphous ZnO_2_ structure occurred at the Cu/ZnO_2_ interfacial region after 3 min of peroxide treatment; the crystalline-to-amorphous phase transformation starts from the surface region of the ZnO_2_ film (ST3; Fig. 1g–i). We believe that the resistivity of an amorphous ZnO_2_ is lesser than that of the crystalline ZnO_2_. Since the ZnO_2_ structure of the ST3 is mainly crystalline, therefore, the resistivity remains high (Fig. 2e). Conversely, the crystalline-to-amorphous phase transformation occurred in almost all regions of the ST9 film (Fig. 1j–l); thus, it leads to a slight decrease in resistivity (Fig. 2f). It is suggested that the number of grain boundaries has more significant role than the thickness parameter in determining the resistivity of ZnO film; higher number of the grain boundaries resulted in lower leakage current [40]. Therefore, we assume that the mechanism of the decreasing resistance phenomenon in the amorphous ZnO_2_ may be similar to the ZnO case which the decreasing number of grain boundaries decreases the resistivity. Nonetheless, a detailed study on the electrical properties of the ZnO_2_ material is an interesting topic that should be explored in the future.

The increase of pristine resistance is beneficial to activate the switching characteristics at lower current compliance (CC) as well as to reduce the operation current of the device. The activation of the switching characteristics is needed to change the pristine state into the low-resistance state (LRS), called as forming. Figure 2g shows the forming process of the fabricated devices. It is shown that the control device requires a very high CC of 100 mA for the forming process; conversely, ST1, ST3, and ST9 devices only require 200, 100, and 35 μA, respectively. It is found that the forming voltage of the devices made with a longer peroxide treatment tends to increase due to the increase in the total thickness of the resistive layer.

**Fig. 3:**
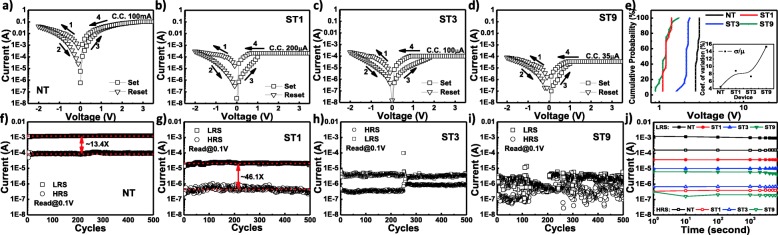
Typical I–V curves of (**a**) control, (**b**) ST1, (**c**) ST3, and (**d**) ST9 devices. **e** Cumulative probability plot of set voltage (Vset). Endurance characteristics of (**f**) control, (**g**) ST1, (**h**) ST3, and (**i**) ST9 devices. **j** Room temperature retention characteristics of all devices. Inset in (**e**) shows the coefficient of variation of the Vset distribution. Each data point in (**e**) represents the 25 consecutive cycles

Figure 3 shows the I–V curves and endurance characteristics of the fabricated devices. All devices exhibit analog counter-clockwise bipolar switching, as shown in Fig. 3a–d. After the forming process, the devices can be switched to the high-resistance stance (HRS) by sweeping the negative voltage bias, called as reset. The reset voltage (Vreset) of all devices is − 2 V. Hereafter, the devices can be switched back to the LRS by sweeping the positive voltage (Vset) bias called as set. The statistical dispersion of Vset may elucidate the relationship between the switching parameter and the switching behavior; [11] thus, a cumulative probability is plotted as shown in Fig. 3e. It is found that the coefficient of variation (standard deviation (σ)/mean (μ)) tends to increase as the time of peroxide treatment increases, as shown in the inset of Fig. 3e. This indicates that the peroxide treatment modulates the switching parameter due to the modification of the shape or size of the conducting bridge [4, 41]. In order to evaluate the device reliability, an endurance test was conducted, and the result is shown in Fig. 3f–i. The control device exhibits very stable switching with ON/OFF ratio (memory window) of approximately 13 times during endurance test, as shown in Fig. 3f. Even though the control device shows good uniformity and sufficient memory window [42], however, the operation current (100 mA) is too high; which is not suitable for low power application [43]. The switching characteristics are enhanced after 1 min of peroxide treatment (ST1), as shown in Fig. 3b and g. The ST1 device is able to operate at much lower operation current (with CC of 200 μA) and exhibits sufficient uniformity with an enlarged memory window of approximately 46 times. Further increase of peroxide treatment time allows the devices to operate at even lower operating current; the ST3 and ST9 devices are able to operate at CC of 100 and 35 μA, respectively, as shown in Fig. 3c and d. Note that the employment of higher CC for ST3 and ST9 may result in device breakdown. Despite both ST3 and ST9 devices operate at much lower current as compared to ST1, the switching uniformity degrades as the time of peroxide treatment increases, as depicted in Fig. 3h and i. Nevertheless, all peroxide-treated devices exhibit an excellent non-volatility behavior, as shown in Fig. 3j; no significant fluctuation is observed for more than 7000 s at room temperature. Based on our previous study, the switching instability is the result of the reduction-oxidation (redox) competition between the multi- and branch conducting bridges [10, 12, 41]. We believe that the formation of the non-confined bridges is significantly controlled by the microstructure of the resistive layer.

**Fig. 4:**
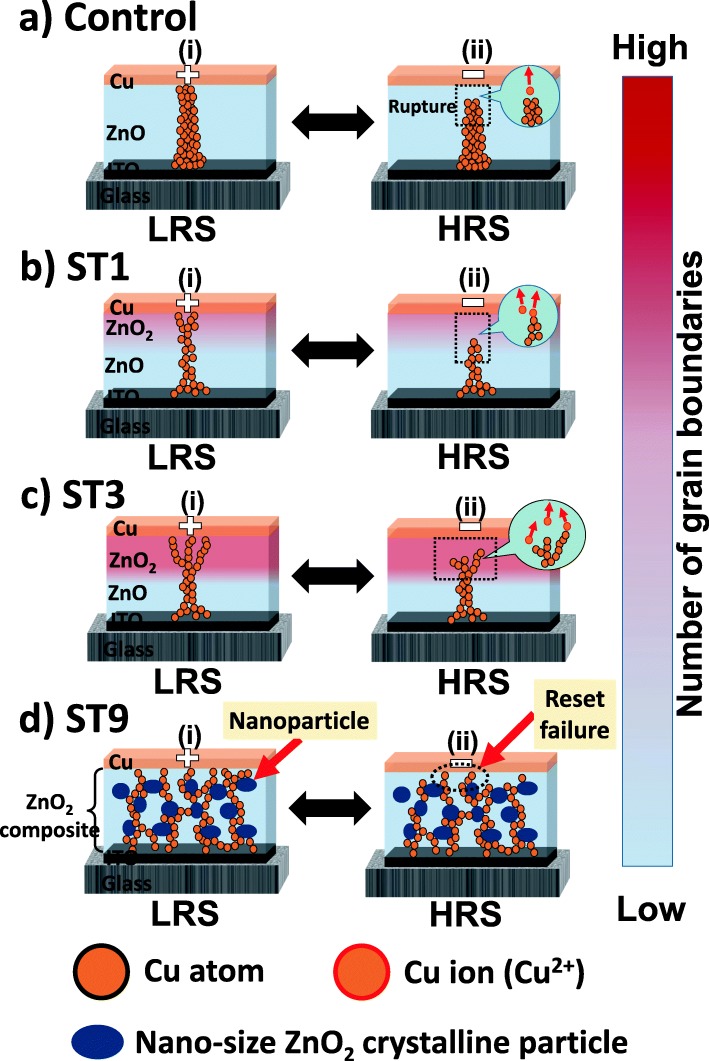
Schematics of conduction mechanism of (**a**) control, (**b**) ST1, (**c**) ST3, and (**d**) ST9 devices

Figure 4a–d shows the schematics of the conduction mechanism of the control device, ST1, ST3, and ST9, respectively. During forming and set processes, the Cu metal is oxidized when a positive bias is applied to the Cu top electrode (TE), and the Cu ions are attracted to the ITO bottom electrode (BE) in order to reduce to the metallic state [8]. This process results in the formation of a conducting bridge that grows from BE to the TE; consequently, the electron can easily flow from cathode to anode and resulted in the LRS (Fig. 4a (i)). Hereafter, the employment of a negative bias to the TE during reset process results in the re-ionization of Cu conducting bridge, and the Cu ions drift back to the TE; hence, the conducting bridge is ruptured, and HRS is achieved (Fig. 4a(ii)). Since the Cu ions tend to drift along the grain boundaries under an electric field [22], therefore, the perpendicular grain orientation of the ZnO resistive layer of the control device (Fig. 1b) helps the formation and rupture of a confined bridge [8]. A confined bridge is beneficial for ensuring that the formation and rupture of the conducting bridge occur at the same region; thus, high-switching uniformity is exhibited in the control device (Fig. 3f). However, the employment of high CC (100 mA) results in the formation of a large conducting bridge and high-current operation. On the other hand, the switching stability for parts of ST1 and ST3 devices degrades (Fig. 3g and h) due to the development of irregular grains (results in higher number of grain boundaries) (Fig. 1e and g). The random microstructure of the ZnO_2_ layer promotes the formation of multi- or branch bridges at the respective region. Since the major area in the ST1-resistive layer is highly perpendicular to ZnO film, therefore, the formation of multi- or branch bridges can be limited (Fig. 4b(i)). Consequently, the degradation of the switching stability is minor, and good endurance performance without any intermediate state (data error) is exhibited (Fig. 4b (ii)). Conversely, a significant area of the randomly oriented ZnO_2_ in the resistive layer of the ST3 device dictates the shape of the conducting bridge and results in the formation of multi- or branch bridges (Fig. 4c (i)). Hence, the formation and rupture may not occur in the same region and leads to a more serious switching instability (Fig. 4c (ii)). For the ST9 case, even though the switching layer has a low number of grain boundaries due to the crystalline-to-amorphous phase transformation, however, the random distribution of the crystalline nanoparticles leads to a severe structure irregularity. Note that since the nanoparticles are in the form of oxide, thus, no enhancement of high electric field around the particle to promote the confinement of the conducting bridge like metal inclusion does [44, 45]. Consequently, the Cu ions drifted randomly, and branched-bridge across the resistive layer is formed during forming and set processes (Fig. 4d (i)). Hereafter, the formation and rupture processes cannot be controlled at the same branch (or region) and results in the set and reset failures (Fig. 4d (ii)); thus, a severe switching instability is exhibited (Fig. 3i).

## Conclusion

In summary, a switching failure mechanism in ZnO_2_-based PMC devices has been proposed. The peroxide treatment promotes the formation of conducting bridge at much lower current compliance due to the high-resistivity of the switching layer. The resistance value of pristine surface-treated device can be increased up to 5 to 6 order of magnitudes. However, an excessive peroxide treatment leads to an increase structural irregularity in the switching layer; thus degrading the switching stability. This suggests that, in fact, the peroxide treatment is a useful method for obtaining low-power PMC devices; however, careful tuning of peroxide treatment is necessary to achieve good switching characteristics. The potential of this technique includes a simple fabrication process flow, scaling down the RRAM structures, and decreasing operation current/power consumption of RRAM devices. Our simple method can be easily adopted (or explored) for many kinds of oxide systems and can encourage the realization of RRAM devices for future non-volatile memory.
